# Association between working alliance and treatment outcomes in a mobile health intervention with a conversational agent (CanRelax)

**DOI:** 10.1016/j.invent.2026.100929

**Published:** 2026-03-13

**Authors:** Sonja Schläpfer, Jürgen Barth, Priska Heinz, Ulrike Held, Tobias Kowatsch, Claudia M. Witt

**Affiliations:** a Complementary and Integrative Digital Health, Institute of Primary Care – University of Zurich and University Hospital Zurich, Switzerland; b Department of Biostatistics at the Epidemiology, Biostatistics and Prevention Institute, University of Zurich, Zurich, Switzerland; c Institute for Implementation Science in Health Care, University of Zurich, Zurich, Switzerland; d School of Medicine, University of St. Gallen, St. Gallen, Switzerland; e Department of Management, Technology, and Economics, ETH Zurich, Zurich, Switzerland

**Keywords:** Digital health, Working alliance, Therapeutic alliance, Conversational agent, Chatbot, Prediction, Distress

## Abstract

This study explored the association between the digital working alliance and treatment outcomes in users of a fully automated mobile health app with a conversational agent (CanRelax app 2.0), which was effective in reducing distress in adults with cancer. This expands research about the working alliance association to an innovative setting. Drawing on secondary data from a randomized controlled trial, we examined whether the strength of the working alliance—and its three subcomponents: bond, task, goal—assessed at week 4 using the Working Alliance Inventory for Internet Interventions, was related to distress levels at week 10. The analysis population consisted of adults diagnosed with cancer in the past five years who received the CanRelax app 2.0 (*N* = 117). Participants accessed a conversational agent–guided intervention featuring mindfulness, relaxation, and personalized weekly coaching modules with behavior change techniques. Mean subscale scores (bond: 4.04, SD = 0.95; goal: 3.46, SD = 0.74; task: 2.79, SD = 0.75; 1–5 scale) indicated good working alliance. Partial correlations adjusting for baseline distress, sex, age, and treatment expectation indicated a small negative association between working alliance and distress (*r* = −0.18), particularly for the task and goal components. No significant association was found for the bond subscale in the overall sample. Associations were stronger in females, and baseline treatment expectations were positively related to working alliance. These findings suggest a stronger working alliance may contribute to better treatment outcomes in fully automated digital health interventions, and optimizing goal and task agreement could further enhance intervention effectiveness.

**Trial registration:**

German Clinical Trials Register (DRKS00027546) on 23.02.2022.

## Highlights

• Users can form a working alliance with conversational agent-guided interventions.
• Stronger alliance is linked to better treatment outcomes.
• Goal and task agreement components show the strongest association with outcomes.
• Optimizing goal and task agreement may enhance digital intervention effectiveness.
• User expectations may influence working alliance development.

## 1 Introduction

The digital working alliance refers to the collaborative relationship between a user and a digital health intervention, such as a smartphone app, web-based program, or conversational agent (Goldberg et al., 2022). It builds on the well-established concept of the working alliance in face-to-face psychotherapy, which comprises agreement on goals, consensus on the tasks necessary to achieve these goals, and the development of a supportive bond (Goldberg et al., 2022; Bordin, 1979; Flückiger et al., 2018). While the working alliance robustly predicts treatment outcomes in face-to-face therapy (Flückiger et al., 2018), its predictive value in digital interventions has been less often investigated. Emerging evidence suggests that the task and goal components may be more strongly associated with outcomes than the bond (Berger, 2017; Probst Greta H et al., 2022; Haaf et al., 2024; Jasper et al., 2014; Ashur et al., 2024). Overall, the working alliance can be regarded as an important working mechanism in digital health interventions as well (Vernmark et al., 2019).

One specific area where the digital working alliance remains unexplored is in the context of conversational agents or chatbots. While research on these interventions is still limited, there is emerging evidence supporting their relevance, particularly from studies on blended digital health interventions in mental health. For example, the strength of the working alliance with conversational agents (e.g., Wysa, Woebot) has been found to be comparable to that of face-to-face interventions (Darcy et al., 2021a). However, robust studies examining the predictive value of the working alliance on health outcomes in rule-based conversational agents are lacking, and recent research on conversational agents using artificial intelligence (AI) has not explored this association, despite having data on the working alliance (Heinz et al., 2025).

To date, most studies on digital working alliance have been conducted in the mental health domain, highlighting the need to investigate the association between digital working alliance and treatment outcomes in other domains. One such domain is cancer care, where the working alliance has rarely been studied in the context of supportive digital interventions. Although a higher level of working alliance has been reported in face-to-face psychosocial care compared to a digital intervention (Flix-Valle et al., 2025), studies suggest that the working alliance in both settings predicts well-being among cancer patients (Bisseling et al., 2019). However, there are also studies where no association was found between the working alliance and health outcomes in cancer care (Nissen et al., 2021).

In light of these mixed findings, the aim of this study is to further investigate the association between the digital working alliance and treatment outcomes. We focus on a fully automated mobile health app featuring a text-based conversational agent designed to reduce distress in adults with cancer (CanRelax app 2.0). The conversational agent offered weekly coaching sessions to motivate participants to practice self-care exercises (e.g., mindfulness and relaxation). This secondary data analysis is based on observational data from a randomized controlled trial (RCT) (Barth et al., 2025; Schläpfer et al., 2024a, Schläpfer et al., 2024b). The CanRelax app 2.0 was effective in reducing distress after 10 weeks (Barth et al., 2025), and the relaxation exercises also lowered perceived distress in the short term (Schläpfer et al., 2024a). In this paper, we examine if and how the digital working alliance – both overall and across its three components (bond, task, and goal) – assessed at week 4, relates to the primary outcome of distress measured at week 10. The primary analysis of the association between the overall working alliance and the primary outcome can be considered confirmatory, whereas all other analyses (e.g., subscale and sex-stratified analyses) are exploratory.

## 2 Materials and methods

### 2.1 Study design

The data for this study were derived from an RCT involving adults with cancer who had high baseline distress. Participants with a score of at least 5 on the Distress Thermometer (Mehnert et al., 2006) were randomly assigned to either the app intervention group or to a waitlist control group. In addition, a nonrandomized comparison arm was included, comprising participants with low baseline distress (less than 5 on the Distress Thermometer). The intervention and nonrandomized groups received immediate access to the app, while the waitlist control group had restricted access for data entry during the first 10 weeks and received full intervention access thereafter. Data from all three groups were pooled for this analysis, as all participants received the same intervention. All participants gave electronic informed consent through the app before enrollment. The study was conducted in accordance with the Declaration of Helsinki and registered at the German Clinical Trials Register (DRKS00027546, 23 February 2022). We submitted the study synopsis to the Ethics Committee of Zurich, Switzerland, and after review, they stated that the study does not fall under the regulation of the Human Research Act of Switzerland (Ethics ID: 2021-01071).

### 2.2 Participants

We enrolled adults (aged ≥18 years) diagnosed with cancer within the past five years, regardless of cancer type or stage. All data were self-reported via the app, and no direct contact with the study team was required throughout the study. Exclusion criteria were suicidal ideation (with provided resources for support, such as contact information for a physician or helpline), insufficient proficiency in German, self-reported pregnancy, or other factors preventing participation (e.g., lack of smartphone knowledge or regular internet access). All participants continued usual care as needed.

### 2.3 Intervention

The CanRelax app 2.0 is a fully automated, theory- and evidence-based digital health intervention designed to promote relaxation and mindfulness. It offers seven guided exercises either audio or biofeedback-based, that were selected by the participants themselves. The app also includes personalized weekly coaching sessions delivered through a rule-based conversational agent, called “Lumy”, and incorporates 39 behavior change techniques (BCTs). Every two weeks, participants set their own goals regarding the number of exercises they wished to practice in the upcoming weeks. To build a strong bond, Lumy was designed to express empathy and respect, provide personalized feedback, celebrate achievements, and offer support in mastering challenges. Lumy incorporated user-specific details (e.g., nickname, pronouns), adapted content to context (e.g., time of day, season), tailored BCTs to individual needs, and continuously personalized sessions based on previous interactions. The app was developed using an iterative design process, ensuring continuous integration of user feedback, and is described in detail in previous publications (Barth et al., 2025; Schläpfer et al., 2024b).

### 2.4 Measures

All outcomes were measured in a standardized manner within the CanRelax app 2.0. The primary outcome was distress assessed 10 weeks after intervention start using the Patient Health Questionnaire Anxiety and Depression Scale (PHQ-ADS (Kroenke et al., 2016)), with the suicidal ideation item excluded. The total score ranged from 0 to 45, with higher scores indicating greater distress.

At week 4, we administered the Working Alliance Inventory for Internet Interventions (WAI—I) (Gómez Penedo et al., 2020) in German. The 12 WAI-I items were adapted from the German version of the WAI-SR (Wilmers et al., 2008; Munder et al., 2010) to suit internet interventions. We customized the items to fit our mobile health study, using the term “CanRelax app” for task and goal items, and the conversational agent's name (“Lumy”) for the bond items (see Appendix A). The scale ranged from 1 to 5, with higher mean scores indicating a stronger working alliance. Internal consistency for the subscales was as follows: bond α = 0.86, task α = 0.83, goal α = 0.77, and for the total score α = 0.91.

At baseline, we collected sociodemographic information, including age and sex assigned at birth (categorized as male or female), using a structured in-app questionnaire. Additionally, we assessed participants' expectations regarding the potential benefits of the CanRelax app 2.0 using the Expectation for Treatment Scale (ETS) (Barth et al., 2019). The ETS consists of five items, measuring anticipated symptom reduction and improvements in functioning, coping, and vitality. Higher scores indicate stronger expectations, with sum scores ranging from 5 to 20 (internal consistency was α = 0.84).

### 2.5 Recruitment and procedures

The app was released in July 2022 on the Apple App Store and Google Play Store, available in Switzerland, Germany, and Austria. Our study website provided key details such as eligibility criteria, app screenshots, and audio samples, along with QR codes for downloading the app. To recruit participants from these countries, we used a combination of social media platforms (LinkedIn, X, Facebook) and traditional methods (consultations with healthcare providers, printed flyers, newsletters, and a press release from the University Hospital Zurich). The app was free of charge, and participants initiated the onboarding process upon downloading it. They were transparently informed that they were interacting with a chatbot rather than a human. The entire study process was fully automated, including screening, consent, enrollment, and data collection. Participants had no direct contact with the research team unless they reached out for technical support.

### 2.6 Data analysis

Participants with available data for the working alliance, the primary outcome (PHQ-ADS at week 10), and baseline variables (PHQ-ADS, sex, age, and ETS) were included in the analyses. Descriptive statistics included mean and standard deviation (SD) or median and interquartile range (IQR) for continuous variables, as well as number and percentage of total for categorical variables. A correlation matrix was computed for all variables used in subsequent analyses—including baseline measures, working alliance, outcome measures, and change in PHQ-ADS scores (defined as the difference between week 10 and baseline PHQ-ADS scores, with negative scores indicating improvement of symptoms)—for the total sample and separately by sex. The primary analysis, conducted on the full sample, consisted of calculating partial correlations in two ways: model 1 estimated the partial correlation between working alliance and the primary outcome, controlling for baseline PHQ-ADS scores; model 2 further adjusted for sex, age, and expectation. Secondary analyses repeated these procedures for each of the three subscales (bond, task, and goal) and were conducted separately by sex. All analyses were conducted in R (version 4.5.1) (R Core Team, 2024).

## 3 Results

### 3.1 Descriptive analyses

Data from 277 datasets were used for this analysis A comparison of baseline characteristics between included participants (*n* = 117) and those who were excluded due to insufficient follow-up data (*n* = 160) is presented in Appendix C. It is apparent that the samples did not differ meaningfully in any of the baseline characteristics. The mean age of the included 117 study participants was 56.0 years (SD 8.9), and the majority were female (98; 83.8%). Most participants were currently employed (78; 66.7%) and had completed tertiary education (76; 64.9%). Breast cancer was the most common diagnosis (47; 40.2%); 27 participants (23.1%) had multiple cancer diagnoses. The average time since diagnosis was 2.1 years (SD 2.7), and 78.6% (92) were currently undergoing cancer therapy (chemotherapy, radiation, or surgery). At baseline, the mean PHQ-ADS score was 16.2 (SD 6.7), indicating moderate levels of anxiety and depression, which decreased to 11.9 (SD 6.9) at week 10. The mean ETS score at baseline was 11.9 (SD 3.0).

Descriptive statistics for the WAI-I total and subscale scores are presented in Table 1. Scores were similar across sexes, with only minor differences between females and males. Among the subscales, the bond subscale had the highest mean score in all groups. Boxplots illustrating the distribution of WAI-I total and subscale scores are provided in Appendix B (Supplementary Figs. B.1 and B.2, with a sex-stratified analysis). The bond subscale showed more variability and was skewed toward the upper end with many participants scoring close to the maximum. In contrast, the task and goal subscales showed less variability.

**Table 1:** Working Alliance Inventory total and subscale scores.

Scale		Full sample N = 117	Female subgroup n = 98	Male subgroup n = 19
Total	Mean (SD)	3.43 (0.71)	3.44 (0.71)	3.37 (0.72)
	Median (IQR)	3.50 (2.92, 4.00)	3.50 (2.92, 4.00)	3.42 (2.92, 4.00)
Task	Mean (SD)	2.79 (0.75)	2.80 (0.74)	2.76 (0.80)
	Median (IQR)	2.75 (2.25, 3.25)	2.75 (2.25, 3.25)	2.75 (2.25, 3.50)
Goal	Mean (SD)	3.46 (0.74)	3.48 (0.73)	3.33 (0.77)
	Median (IQR)	3.50 (3.00, 4.00)	3.50 (3.00, 4.00)	3.50 (3.00, 3.75)
Bond	Mean (SD)	4.04 (0.95)	4.05 (0.97)	4.01 (0.86)
	Median (IQR)	4.25 (3.25, 5.00)	4.25 (3.25, 5.00)	4.00 (3.25, 5.00)

Note. Measured with the German version of the Working Alliance Inventory for Internet Interventions (WAI-I).

**Fig. 1:**
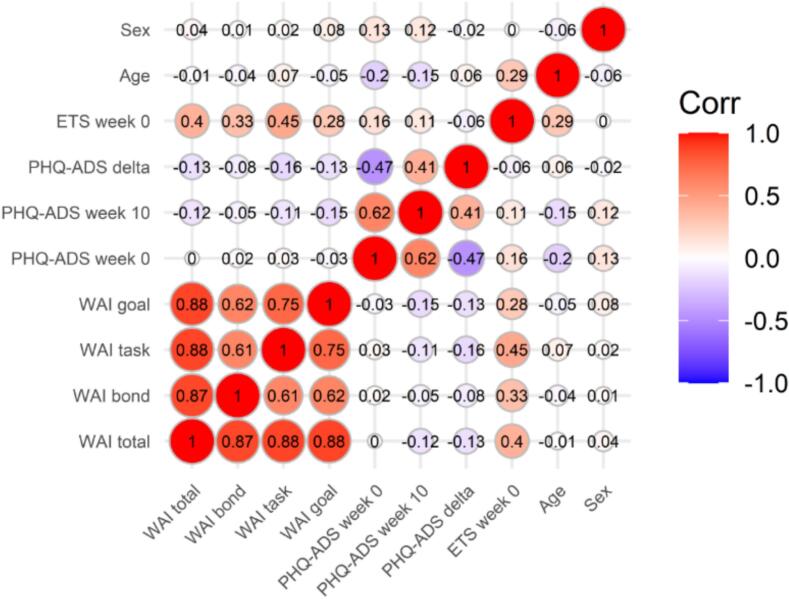
Bivariate correlation matrix for all variables in the full sample (*N* = 117). Red indicates a positive association, while blue indicates a negative association between the variables. Note. WAI = working alliance inventory (total or subscales bond, task, and goal); PHD-ADS = anxiety and depression scale score at baseline (week 0), at week 10 (primary outcome), or the change from baseline to week 10 (delta); ETS = expectation at baseline; sex (0 = male, 1 = female).

The correlation matrix for all variables used in the analyses, including working alliance total and subscales, PHQ-ADS scores at baseline, week 10, and their change (delta), sex, age, and expectation, is shown for the full sample in Fig. 1, with separate matrices for females and males provided in Appendix B (Supplementary Figs. B.3 and B.4). The matrix indicated small negative correlations between working alliance and distress at week 10, as well as between working alliance and change in distress, but no correlation between baseline distress and working alliance. This pattern was consistent among female participants. In contrast, the results for males differed. Working alliance demonstrated a small to moderate positive correlation with baseline distress, which might affect further analyses, as a suppressor effect could be present. In fact, working alliance also showed small positive correlations with distress at week 10, indicating that participants with higher working alliance had higher distress. Correlations between working alliance and change in distress among males ranged from negligible positive associations for the bond subscale to moderate negative associations for the goal subscale. Additionally, working alliance was consistently and positively associated with expectation at baseline, both in the full sample and across sexes. Age and sex were not associated with working alliance.

### 3.2 Primary and secondary analyses

Partial correlations between the total working alliance score and PHQ-ADS at 10 weeks (adjusted for baseline distress, sex, age, and expectation) showed a small negative association (partial *r* = −0.18, 95% CI [−0.35, 0.00]; Table 2). Similar negative associations were observed for the task and goal subscales (task: *r* = −0.20, 95% CI [−0.37, −0.01]; goal: *r* = −0.19, 95% CI [−0.37, −0.01]), while the bond subscale was not significantly related to distress overall. Sex-stratified analyses indicated that these negative associations were present in females but not in males. Notably, the bond subscale showed a small positive partial correlation with distress in males, although with substantial uncertainty (*r* = 0.11, 95% CI [−0.41, 0.57]), contrasting with the negative or null correlations observed in females and the full sample.

**Table 2:** Partial correlations between working alliance and distress at week 10.

Predictor	Model	Full sample N = 117		Female subgroup n = 98		Male subgroup n = 19	
		Partial r	95%-CI	Partial r	95%-CI	Partial r	95%-CI
Total	Model 1	−0.15	[−0.32, 0.03]	−0.17	[−0.36, 0.03]	−0.02	[−0.48, 0.45]
Total	Model 2	−0.18	[−0.35, 0.00]	−0.20	[−0.38, 0.01]	−0.06	[−0.54, 0.45]
Task	Model 2	−0.20	[−0.37, −0.01]	−0.22	[−0.40, −0.01]	−0.09	[−0.56, 0.43]
Goal	Model 2	−0.19	[−0.37, −0.01]	−0.19	[−0.38, 0.01]	−0.22	[−0.64, 0.32]
Bond	Model 2	−0.10	[−0.27, 0.09]	−0.12	[−0.31, 0.09]	0.11	[−0.41, 0.57]

Note. To examine the association between the working alliance and the primary outcome (PHQ-ADS at week 10), partial correlations were calculated in two models. The first model adjusted for baseline distress (PHQ-ADS at baseline; covariate PHQ-ADS-0), while the second model adjusted for both baseline distress and additional covariates, including sex, age, and expectation (ETS; covariates PHQ-ADS-0, Sex, Age, ETS). Both analyses were conducted using the population with available data on the working alliance, outcome measures, and baseline variables (PHQ-ADS, sex, age, ETS).

## 4 Discussion

Our findings indicate a small but interesting association between higher working alliance and lower distress at 10 weeks, with about 5% of the changes in the health outcome attributed to variability in the working alliance. This association was consistent across the subscales of the working alliance in the full sample and in females, but not in the much smaller group of males.

The observed association between higher working alliance and reduced distress is supported by meta-analytic evidence for an alliance-outcome relationship in digital interventions (Flückiger et al., 2018; Probst Greta H et al., 2022; Haller et al., 2023; Kaiser et al., 2021). However, both our findings and those of previous studies suggest that the strength and consistency of this association can vary, especially in unguided digital interventions (Goldberg et al., 2022; Cavanagh and Millings, 2013; Clarke et al., 2016). In this context, our results are particularly noteworthy, as they demonstrate that a meaningful working alliance can emerge even in a fully automated intervention with a conversational agent.

Participants generally evaluated the working alliance with the CanRelax app 2.0 positively, similar to previous studies with rule-based conversational agents (Kowatsch et al., 2021a, Kowatsch et al., 2021b), though differences emerged across the three subscales. The bond subscale had the highest mean score, comparable to scores reported in research on free-text conversational agents using artificial intelligence (Beatty et al., 2022; Darcy et al., 2021b), and thus contrasting Mai, Neef and Richert (Mai et al., 2022), who reported higher bond scores for free-text agents than for rule-based ones. Our high bond score indicates that participants tended to feel emotionally supported and understood by the conversational agent Lumy, likely reflecting the emphasis on relational aspects in Lumy's design.

While the bond subscale had the highest mean score, the task and goal subscales were more strongly associated with reductions in distress at the end of the intervention. This pattern is consistent with earlier findings showing that task and goal aspects may be more predictive of outcomes than bond in digital interventions (Berger, 2017; Probst Greta H et al., 2022), supporting ongoing efforts to adapt the working alliance concept for digital contexts (Goldberg et al., 2022; Ashur et al., 2024; Malouin-Lachance et al., 2025; Henson et al., 2019). It also aligns with findings from a recent meta-analysis on the digital working alliance in guided interventions, which showed that the task subscale was generally more predictive of treatment outcomes than bond (Probst Greta H et al., 2022; Miloff et al., 2020; Bur et al., 2022). Similarly, studies on unguided digital interventions have identified the task subscale as the strongest predictor of outcomes (Haaf et al., 2024; Jasper et al., 2014).

One possible explanation for the weaker association between bond and outcomes is the ceiling effect we observed on the bond subscale (see Supplementary Fig. B.1). Similar ceiling effects and skewed bond data have been documented in other studies, which may statistically limit the detection of meaningful associations with outcomes (Berger, 2017; Jasper et al., 2014; Knaevelsrud and Maercker, 2006; Berger et al., 2014). A second explanation could be that, similar to face-to-face interventions, agreement on goals and tasks forms the basis of successful treatment, whereas a stronger bond may be a consequence of early improvements in health rather than a predictor of health outcomes (Webb et al., 2011). Third, previous research has identified emotion regulation (Lindqvist et al., 2023) and self-efficacy (Burns and Evon, 2007) as working mechanisms in the alliance-outcome association. Both constructs can be considered coping resources, which might be more strongly related to task and goal components.

We also observed noteworthy differences between female and male participants. Female participants reported slightly higher working alliance scores, consistent with previous findings (Sagui-Henson et al., 2022). Moreover, the association between working alliance and the primary outcome (PHQ-ADS at 10 weeks) was consistently stronger among female participants, as indicated by robust negative correlations. This suggests that the female participants in our study who experienced a stronger working alliance with the CanRelax app also reported lower distress at the end of the intervention. In contrast, these associations were weaker or absent in male participants. The bond subscale even showed a weak positive correlation with distress in this group, suggesting that male participants may engage differently with the relational aspects of the CanRelax app. Importantly, female and male participants did not differ in terms of baseline expectations, indicating that sex differences in alliance or outcomes are unlikely to be explained by initial treatment expectations.

Across the full sample, higher baseline expectations were positively associated with higher working alliance scores. This finding aligns with previous research indicating that certain aspects of treatment expectations can predict the development of a stronger working alliance (Patterson et al., 2008; Bergman Nordgren et al., 2013; Tokar et al., 1996). Individuals who begin an intervention with higher expectations may therefore be more likely to perceive a stronger alliance. However, our main analysis adjusted for baseline expectations and still revealed an association between working alliance and outcomes. This suggests that, while expectations play a role, they do not fully explain the observed relationship. Nevertheless, these findings highlight the importance of considering participants' initial expectations when interpreting working alliance scores and their influence on intervention outcomes. They also underscore the potential benefit of actively fostering positive expectations at the outset of digital interventions and point to the value of further research into the complex interplay between expectations, alliance development, and outcomes in digital health settings.

### 4.1 Limitations

Some methodological limitations must be considered when interpreting our findings. First, our analysis only includes participants who completed the study, which may introduce bias. Individuals who dropped out – potentially due to limited treatment effects and who may have had lower working alliance scores –are not represented in our data. As a result, the association between working alliance and outcomes may be underestimated (Flückiger et al., 2013). In addition, most participants were females with breast cancer, which limits the generalizability of the findings to other contexts.

Second, we assessed working alliance only at a single time point (week 4), without considering any reductions in distress that may have occurred prior to this assessment. This raises the possibility that working alliance scores were influenced by early symptom improvement, rather than serving as an independent predictor of treatment outcomes (Elvins and Green, 2008). However, prior research indicates that the working alliance predicts outcomes independently of early symptom improvement (Flückiger et al., 2013), suggesting that its association with treatment outcomes may not be solely attributable to early changes in distress. Therefore, further research with additional time points to measure working alliance and symptoms could help to disentangle the sequence of changes using statistical procedures like cross-lagged panel analysis, growth models, or similar techniques (Vernmark et al., 2019). Additionally, the use of the WAI-I may be subject to limitations, as this measure was originally developed for guided eHealth interventions and may not fully translate to the context of our study. In this setting, users evaluated their relationship with both the conversational agent and the app, which may differ conceptually from working alliances formed with a single entity.

Third, the sex-specific results should be interpreted with caution due to the low number of male study participants. Generalizability is further limited by the high proportion of female participants with breast cancer in our sample.

Finally, we controlled for some relevant variables, but we acknowledge that other potential moderators of treatment effects and associations with working alliance may exist. Variables such as engagement, technical skills, and user experience could also be associated with working alliance and treatment effects.

## 5 Conclusion

Our study contributes to the growing body of evidence that users can establish a working alliance with unguided digital interventions, and that this working alliance is associated with improved treatment outcomes. Specifically, our findings underscore the potential value of optimizing goal and task agreement within digital interventions to further enhance their effectiveness. Finally, participants' expectations might influence working alliance, and their role warrants further investigation in digital intervention research.

## Glossary

Lumy: Rule-based conversational agent that guides users through the CanRelax intervention and delivers personalized weekly coaching sessions

## Abbreviations

ETS: Expectation for Treatment Scale
PHQ-ADS: Patient Health Questionnaire Anxiety and Depression Scale
RCT: Randomized controlled trial
WAI-I: Working Alliance Inventory for Internet Interventions

## CRediT authorship contribution statement

SSC and JB wrote the first draft of the manuscript and, together with PH and UH, developed the statistical analysis plan. SSC, JB, CW, and TK contributed to the development of the app-based intervention and the conduct of the study. PH and UH performed the statistical analyses. All authors critically revised the manuscript and approved the final version.

## Declaration of Generative AI and AI-assisted technologies in the writing process

During the preparation of this work, the authors used ChatGPT (OpenAI) and Perplexity (Perplexity AI, Inc.) to improve the readability and language of the manuscript. After using these tools, the authors reviewed and edited the content as needed and take full responsibility for the content of the published article.

## Funding source

This work was supported by the 10.13039/501100013362Swiss Cancer Research foundation [grant number KFS 4556-08-2018]. The funding source was not involved in the design and conduct of the study, in the collection, analysis, and interpretation of data, in the writing of the report, and in the decision to submit the article for publication.

## Declaration of competing interest

The authors declare the following financial interests/personal relationships which may be considered as potential competing interests: Claudia M. Witt reports financial support was provided by Swiss Cancer Research foundation. Claudia M. Witt reports a relationship with Digitalization Initiative of the Zurich Higher Education Institutions that includes: funding grants. Claudia M. Witt reports a relationship with Swiss Cancer Research foundation that includes: funding grants. Claudia M. Witt reports a relationship with Swiss National Science Foundation that includes: funding grants. Claudia M. Witt reports a relationship with German Health Care Innovation Fund that includes: funding grants. Claudia M. Witt reports a relationship with Newsense Lab GmbH that includes: funding grants. Claudia M. Witt reports a relationship with Swiss hospitals that includes: speaking and lecture fees. Tobias Kowatsch reports a relationship with CSS (Swiss health insurer) that includes: funding grants. Tobias Kowatsch reports a relationship with MTIP (Swiss digital health investor company) that includes: funding grants. Tobias Kowatsch reports a relationship with Mavie Next (Austrian health provider) that includes: funding grants. Jürgen Barth reports a relationship with Diverse that includes: speaking and lecture fees. Developer and promoter of the open-source software platform MobileCoach (TK) Co-founder of Pathmate Technologies, a University spin-off company that creates and delivers digital clinical pathways (not involved in this research; TK) If there are other authors, they declare that they have no known competing financial interests or personal relationships that could have appeared to influence the work reported in this paper.

## Data Availability

Data are available on OSF: https://osf.io/9nqyb/.

## Appendix A Supplementary data

Appendix A. German version of the WAI-I Adapted for the CanRelax App with the conversational agent Lumy. Image 1

Appendix B. Supplementary figures. Image 2

Appendix C Table C1. Comparison of excluded and included participants in baseline characteristics. Image 3
