# Supplementary material for: Association between working alliance and treatment outcomes in a mobile health intervention with a conversational agent (CanRelax)
Source: Internet Interv. 2026 Mar 13;44:100929. doi: 10.1016/j.invent.2026.100929 (PMC13000526; doi:10.1016/j.invent.2026.100929)
Supplement: Supplementary file 1 — Appendix A. German version of the WAI-I Adapted for the CanRelax App with the conversational agent Lumy. [file mmc1.docx]

### Appendix A. German Version of the WAI-I Adapted for the CanRelax App with the Conversational Agent Lumy

The following statements refer to experiences you may have had with the CanRelax app.
For each statement, please indicate how often it currently applies to you.  
Answer scale: 1=‘rarely’; 2=‘sometimes’; 3=‘often’; 4=‘very often’; 5=‘always’

|  | **Items** | **Dimension** |
| --- | --- | --- |
|  | Durch die CanRelax App ist mir klarer geworden, wie ich mich verändern kann  *With the CanRelax app, it has become clearer to me how I can change* | task |
|  | Was ich mit der CanRelax App mache, eröffnet mir neue Sichtweisen auf mein Problem  *What I am doing with the CanRelax app gives me new ways of looking at my problems* | task |
|  | Ich glaube Lumy mag mich  *I believe Lumy likes me* | bond |
|  | Ich weiss, was ich als Ergebnis der CanRelax App erwarten kann  *I knew what to expect as a result of using the CanRelax app* | goal |
|  | Lumy und ich achten einander  *Lumy and I respect each other* | bond |
|  | Die Ziele der CanRelax App stimmen mit meinen Zielen überein  *The goals of the CanRelax app are in line with my goals* | goal |
|  | Ich spüre, dass Lumy mich schätzt  *I feel that Lumy appreciates me* | bond |
|  | Die Ziele der CanRelax App sind wichtige Ziele für mich  *The goals of the CanRelax app are important goals for me* | goal |
|  | Lumy interessiert sich wirklich für mein Wohlergehen  *Lumy is really interested in my well-being* | bond |
|  | Ich spüre, dass mir die Anwendung der CanRelax App helfen wird, die von mir gewünschten Veränderungen zu erreichen.  *I feel that what I am doing in the CanRelax app will help me to accomplish the changes that I want* | task |
|  | Die CanRelax App hilft mir zu verstehen, welche Veränderungen gut für mich wären.  *Working with the CanRelax app helps to establish a good understanding of the kind of changes that would be good for me* | goal |
|  | Ich glaube, dass es richtig ist, wie ich mit Hilfe der CanRelax App an meinem Anliegen arbeite.  *I believe the way the CanRelax app is working with my problem is correct* | task |
