# Supplementary material for: Association between working alliance and treatment outcomes in a mobile health intervention with a conversational agent (CanRelax)
Source: Internet Interv. 2026 Mar 13;44:100929. doi: 10.1016/j.invent.2026.100929 (PMC13000526; doi:10.1016/j.invent.2026.100929)
Supplement: Supplementary file 2 — Appendix B. Supplementary figures. [file mmc2.docx]

### Appendix B. Supplementary Figures

#### Supplementary Figure B.1. Boxplots illustrating the distribution of WAI-I total and subscale scores


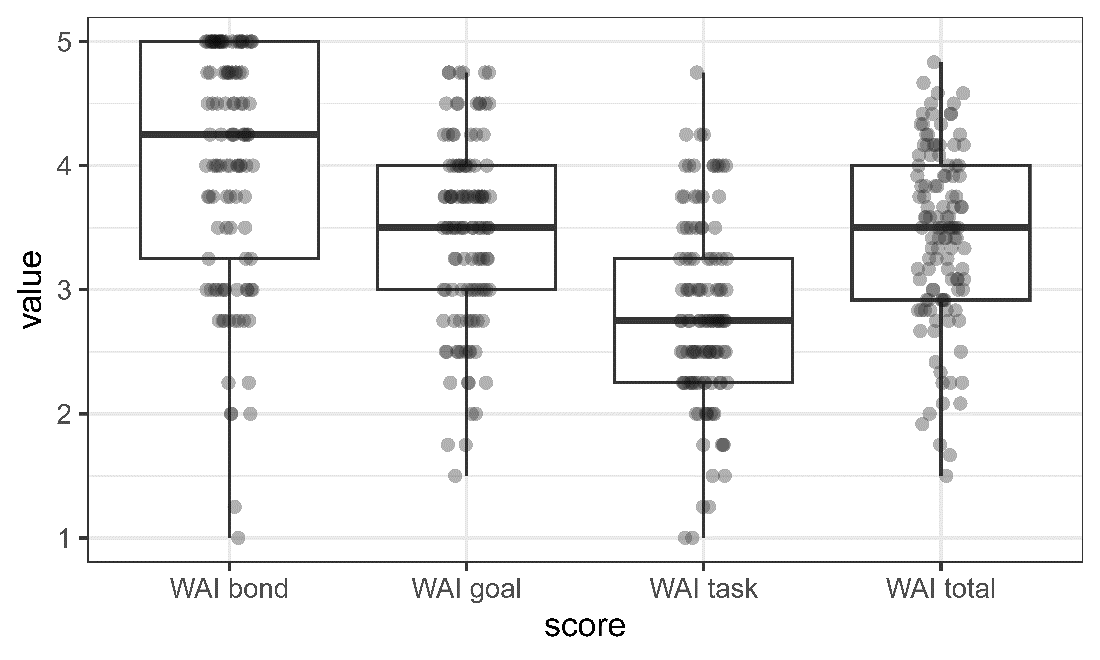


*Note.* WAI = working alliance inventory (total or subscales bond, goal, and task)

#### Supplementary Figure B.2. Boxplots illustrating the distribution of WAI-I total and subscale scores stratified by sex


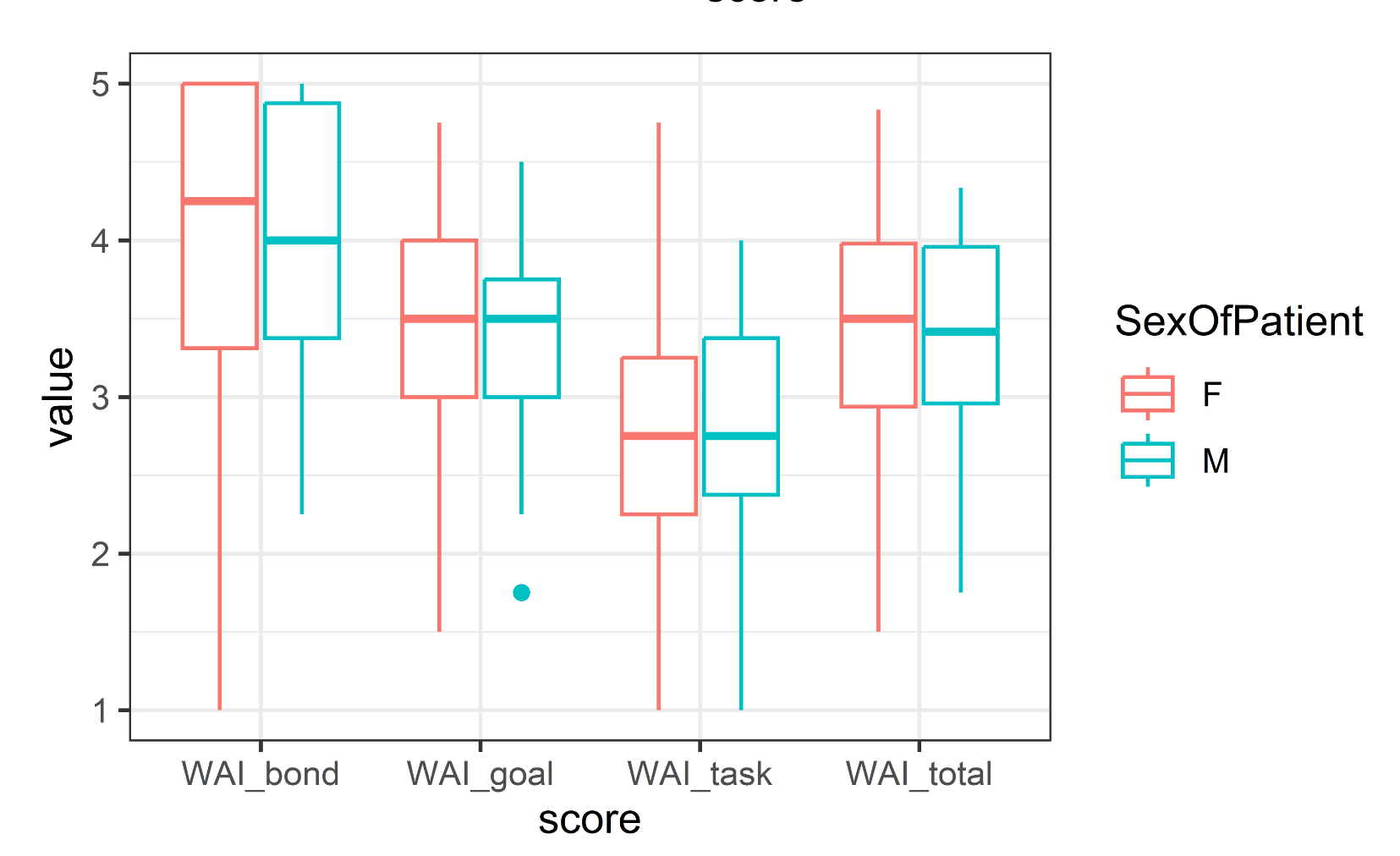


#### Supplementary Figure B.3. Bivariate Correlation Matrix for All Variables in the Female Subsample (n=98)


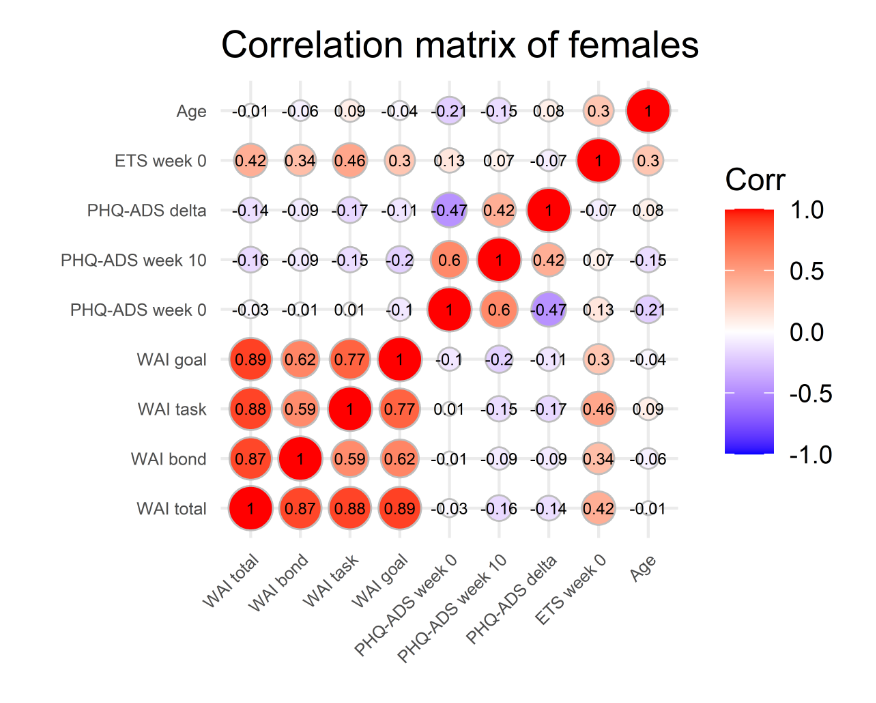


*Note.* WAI = working alliance inventory (total or subscales bond, task, and goal); PHD-ADS = anxiety and depression scale score at baseline (week 0), at week 10 (primary outcome), or the change from baseline to week 10 (delta); ETS = expectation at baseline

#### Supplementary Figure B.4. Bivariate Correlation Matrix for All Variables in the Male Subsample (n=19)


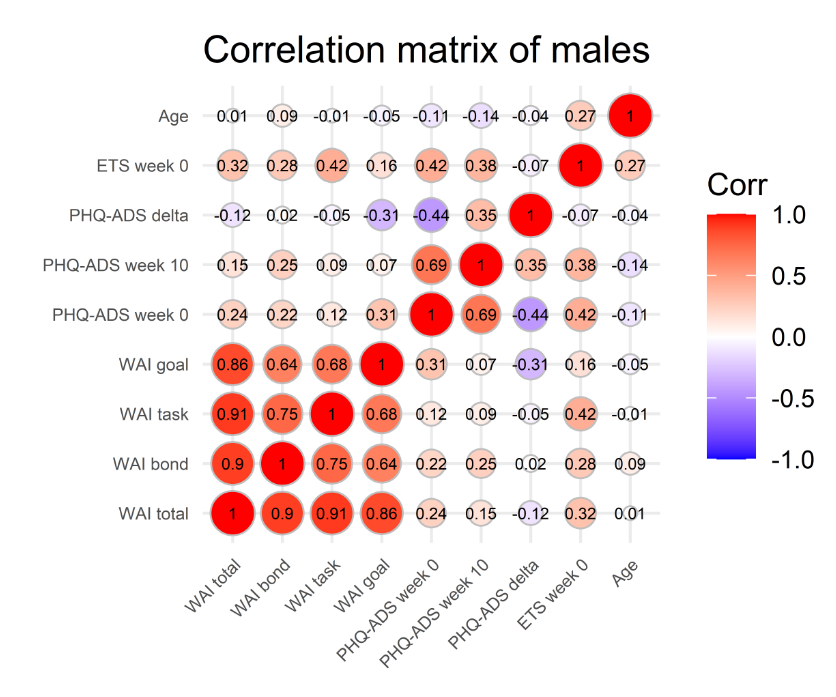


*Note.* WAI = working alliance inventory (total or subscales bond, task, and goal); PHD-ADS = anxiety and depression scale score at baseline (week 0), at week 10 (primary outcome), or the change from baseline to week 10 (delta); ETS = expectation at baseline
