# Supplementary material for: Association between working alliance and treatment outcomes in a mobile health intervention with a conversational agent (CanRelax)
Source: Internet Interv. 2026 Mar 13;44:100929. doi: 10.1016/j.invent.2026.100929 (PMC13000526; doi:10.1016/j.invent.2026.100929)
Supplement: Supplementary file 3 — Appendix C Table C1. Comparison of excluded and included participants in baseline characteristics. [file mmc3.docx]

### Appendix C

#### Table C1: Comparison of excluded and included participants in baseline characteristics

| **Characteristic** | **Overall N=277** | **Excluded n=160** | **Included n=117** |
| --- | --- | --- | --- |
| Group (%) |  |  |  |
| Control group 2 | 67 (24.2) | 35 (21.9) | 32 (27.4) |
| Control group | 105 (37.9) | 70 (43.8) | 35 (29.9) |
| Intervention group | 105 (37.9) | 55 (34.4) | 50 (42.7) |
| Age (mean (SD)) | 54.96 (10.24) | 54.19 (11.08) | 56.00 (8.91) |
| Sex of participant (%) |  |  |  |
| Female | 223 (80.5) | 125 (78.1) | 98 (83.8) |
| Male | 54 (19.5) | 35 (21.9) | 19 (16.2) |
| Education (%) |  |  |  |
| Basic education | 6 (2.2) | 6 (3.8) | 0 (0.0) |
| Middle school | 12 (4.3) | 8 (5.0) | 4 (3.4) |
| Apprenticeship | 67 (24.2) | 41 (25.6) | 26 (22.2) |
| High school or similar | 29 (10.5) | 18 (11.2) | 11 (9.4) |
| Higher education | 54 (19.5) | 28 (17.5) | 26 (22.2) |
| University or similar | 109 (39.4) | 59 (36.9) | 50 (42.7) |
| Employment (%) |  |  |  |
| No | 96 (34.7) | 57 (35.6) | 39 (33.3) |
| Yes | 181 (65.3) | 103 (64.4) | 78 (66.7) |
| Cancer diagnosis (%) |  |  |  |
| Lung | 9 (3.2) | 7 (4.4) | 2 (1.7) |
| Colon | 7 (2.5) | 4 (2.5) | 3 (2.6) |
| Skin | 19 (6.9) | 13 (8.1) | 6 (5.1) |
| Lymphoma | 13 (4.7) | 8 (5.0) | 5 (4.3) |
| Pancreas | 1 (0.4) | 1 (0.6) | 0 (0.0) |
| Bladder | 1 (0.4) | 0 (0.0) | 1 (0.9) |
| Prostate | 4 (1.4) | 3 (1.9) | 1 (0.9) |
| Breast | 96 (34.7) | 49 (30.6) | 47 (40.2) |
| Uterus | 4 (1.4) | 4 (2.5) | 0 (0.0) |
| Other | 50 (18.1) | 25 (15.6) | 25 (21.4) |
| multiple diagnoses | 73 (26.4) | 46 (28.7) | 27 (23.1) |
| WHO5 (mean (SD)) | 44.85 (21.55) | 44.52 (21.41) | 45.30 (21.81) |
| ETS (mean (SD)) | 12.13 (3.11) | 12.33 (3.20) | 11.85 (2.97) |
| Technology affinity  (mean (SD)) | 9.67 (1.98) | 9.73 (2.00) | 9.59 (1.97) |
| PHQ-ADS (mean (SD)) | 17.09 (7.31) | 17.69 (7.68) | 16.26 (6.70) |

WHO-Five Well-Being Index (higher score represents better well-being); ETS Expectation for Treatment Scale (higher scores indicating stronger expectations); Technology affinity (higher scores representing higher technology affinity); PHQ-ADS Patient Health Questionnaire Anxiety and Depression Scale (higher score indicating more distress)

More information is available here: [Mobile health intervention CanRelax reduces distress in people with cancer in a randomized controlled trial | npj Digital Medicine](https://www.nature.com/articles/s41746-025-01688-x)
